# Clinical characteristics of combined rosacea and migraine

**DOI:** 10.3389/fmed.2022.1026447

**Published:** 2022-10-20

**Authors:** Nita K. F. Wienholtz, Casper E. Christensen, Ditte G. Zhang, Anne-Sofie A. Rechnagel, Helene V. S. Byrnel, Jeanette H. Haugaard, Messoud Ashina, Jacob P. Thyssen, Alexander Egeberg

**Affiliations:** ^1^Department of Neurology, Danish Headache Center, Rigshospitalet Glostrup, University of Copenhagen, Copenhagen, Denmark; ^2^Department of Dermatology and Allergy, Herlev and Gentofte Hospital, University of Copenhagen, Hellerup, Denmark; ^3^Department of Dermatology, Bispebjerg Hospital, University of Copenhagen, Bispebjerg, Denmark

**Keywords:** rosacea, migraine, interview, prevalence, overlap

## Abstract

**Background:**

An overlap between the skin disease rosacea and the headache disease migraine has been established; however, the magnitude of this overlap and the distribution between subtypes/phenotypes remains unclear.

**Objective:**

The aim was to determine the magnitude of the overlap between rosacea and migraine, and to determine which subtypes/phenotypes were present in patients with concomitant rosacea and migraine.

**Methods:**

In this cross-sectional study, 604 patients with a diagnosis of either rosacea or migraine were phenotyped through a face-to-face interview with clinical examination, to determine prevalence and phenotype of rosacea, and prevalence and subtype of migraine.

**Results:**

We found a prevalence of migraine of 54% in patients with rosacea, and a prevalence of rosacea of 65% in patients with migraine. Concomitant migraine was significantly associated with the rosacea features flushing (odds ratio = 2.6, 95% confidence interval = 1.4–4.7, *p* = 0.002), ocular symptoms (odds ratio = 2.4, 95% confidence interval = 1.5–3.9, *p* < 0.001), and burning (odds ratio = 2.1, 95% confidence interval = 1.3–3.4, *p* = 0.002), whereas papules/pustules were inversely related with concomitant migraine (odds ratio = 0.5, 95% confidence interval = 0.3–0.8, *p* = 0.006). No association was found between concomitant migraine and centrofacial erythema, rhinophyma, telangiectasia, edema, or dryness. Concomitant rosacea was not associated with any specific migraine subtype in patients with migraine.

**Conclusion:**

This study highlights a substantial overlap between rosacea and migraine, particularly in patients with certain rosacea features. Individuals with rosacea should be asked about concomitant migraine, and comorbidities should be considered when choosing between treatments.

## Key points

– There was a substantial overlap between rosacea and migraine, and more than half the patients with rosacea had concomitant migraine.
– Almost half the patients with concomitant migraine were unaware of their migraine diagnosis.
– Some migraine treatments can worsen rosacea, and it is important to consider comorbidities when choosing between treatment options for rosacea.

## Introduction

Rosacea is a common, chronic inflammatory skin disease affecting 5.5% of the adult population (1). Rosacea has been associated with the headache disease migraine which affects up to 20% globally (2, 3). Rosacea and migraine both primarily affect young individuals of Caucasian descent, and are characterized by relapsing episodes of distinct and debilitating symptoms deriving from the trigeminal innervated area (4–7). Common triggers for both rosacea and migraine include physical and mental stress, certain foods and beverages, ultra violet exposure, heat, and cold (7–9), and both diseases have been associated with anxiety and depression, severely affecting quality of life (10–13). While migraine evidently is a neurovascular condition it seems that certain rosacea features such as flushing, and the neurogenic stinging and burning are attributed to neurovascular alteration and upregulation of signaling neuropeptides such as calcitonin-gene-related peptide and pituitary adenylate cyclase-activating polypeptide-38 (9, 14–17). Epidemiological and clinical studies have shown a positive association between rosacea and migraine (18–20) although the exact magnitude of the overlap and distribution between subtypes/phenotypes remain unclear.

In this cross-sectional interview study based on face-to-face interview and clinical examination, we aimed to determine the overlap between rosacea and migraine as well as determining whether concomitant rosacea and migraine was associated with certain subtypes/phenotypes or severity of each disease.

## Materials and methods

### Study population and design

This was a cross-sectional study based on interviews and clinical examinations conducted between September 2018 and October 2019. Patients were included into two cohorts and enrollment was based on patients diagnosed with either rosacea or migraine who were managed in tertiary care at one of three University hospitals in Copenhagen, Denmark (Danish Headache Center, Rigshospitalet Glostrup; Department of Dermatology and Allergy, Herlev and Gentofte Hospitals; Department of Dermatology, Bispebjerg Hospital). Details on rationale, design, and study procedures have been published elsewhere (21).

### Patients

#### Migraine diagnosis

Patients with rosacea were included in the *Copenhagen Rosacea Cohort* (COROCO). A semi- structured validated interview (22) (Supplementary Data Sheet 1) was used to diagnose and subtype migraine according to the International Headache Classification (4). Migraine diagnosis was defined as at least 5 attacks of headache fulfilling migraine criteria (lifetime prevalence). One-year prevalence was defined as patients fulfilling criteria for lifetime prevalence with *at least* one migraine attack in the past year. Subtype was defined as either migraine *with* or *without* aura. Frequency was collected retrospectively as average migraine attacks per month in the past year (self-reported). Chronic migraine was defined as 15 days of headache or more per month with at least 8 days of migraine and episodic migraine was defined as less than 15 headache/migraine days per month.

#### Rosacea diagnosis

Rosacea was diagnosed and phenotyped based on a semi-structured face-to-face interview (Supplementary Data Sheet 2) with clinical examination supplemented with clinical photographs (evaluated by three experienced physicians: NKFW, JPT, AE) according to the 2017 updated classification (23). For severity, Investigator’s Global Assessment (IGA) (24), Clinician Erythema Assessment (CEA) (25), and the newly developed and validated Rosacea Area and Severity Index [RASI] *(manuscript submitted)* were applied. RASI is an objective index evaluating the four major rosacea features: “erythema, papules/pustules, telangiectasia and phymatous changes” in four facial areas: “cheeks, forehead, nose, chin.” Evaluation of features results in a RASI score between 0 and 72. Clear/almost clear (IGA 0) was defined as > 0–3.0 on RASI; Very Mild Rosacea (IGA 1) was defined as 3.1–5.9 on RASI; Mild Rosacea (IGA 2) was defined as 6.0–9.9 on RASI; Moderate Rosacea (IGA 3) was defined as 10.0–19.9 on RASI; Severe Rosacea (IGA 4) was defined as RASI 20.0 or greater. Subtype/phenotyping of rosacea was based on the 2002 and 2017 guidelines (5, 23). Phymatous rosacea was not included in correlation analyses due to the overall low number of patients with phymatous rosacea in both cohorts.

### Outcome measures and statistical analyses

This was a cross-sectional study based on two cohorts. Due to difference in distribution of age and sex cohorts were not directly compared. Continuous data were presented as means with standard deviations (SD) or medians with ranges, and categorical data as numbers with percentages. Rosacea phenotype in patients with/without concomitant migraine, and migraine subtype in patients with/without concomitant rosacea were assessed with multivariate logistic regressions adjusted for age, sex, and smoking, and odds ratios (OR) were calculated. All tests were considered statistically significant at *P*-value < 0.05. Statistical analyses were performed in SAS Enterprise Guide 7.1 (SAS Institute Inc.).

## Results

### Patient characteristics

The COROCO included 300 patients with rosacea (203 women and 97 men) with a mean age of 50.2 years (*SD* = 12.9). Prevalence of migraine was 54% (163 patients). Mean age at onset of rosacea was 26.6 years (*SD* = 13.4 years) and mean age at onset of migraine was 24.3 years (*SD* = 16.3 years).

The COMICO included 304 patients with migraine (269 women and 35 men) with a mean age of 40.8 years (*SD* = 12.9). Prevalence of rosacea was 65% (196 patients). Mean age at onset of migraine was 24.0 years (*SD* = 17.3) and mean age at onset of rosacea was 36.7 years (*SD* = 14.6).

**FIGURE 1:**
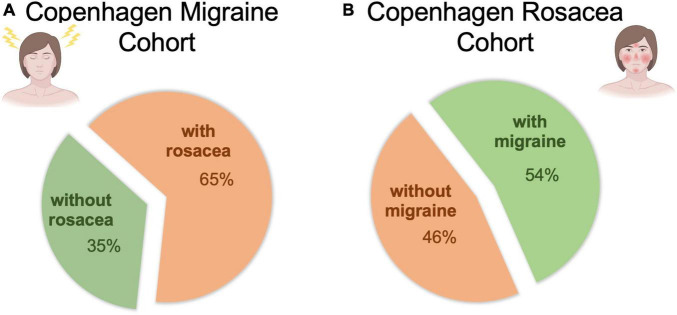
Prevalence of rosacea and migraine in each cohort. **(A)** Prevalence of rosacea in COMICO. **(B)** Prevalence of migraine in COROCO. COMICO, Copenhagen Migraine Cohort; COROCO, Copenhagen Rosacea Cohort.

For details on enrollment and demographics, see Figure 1, Supplementary Figure 1 and Supplementary Table 1.

### Migraine

#### Prevalence and subtype of migraine

In COROCO, we found an overall prevalence of migraine of 54%, and a 1-year prevalence of 41% (52% in women, 19% in men). The 1-year prevalence peaked at 55% between ages 25 and 54 years for women, whereas prevalence was similar for men at all ages (∼30%). Of those with migraine in COROCO, 131 patients (68%) had migraine *without* aura (MO), 61 patients (32%) had migraine *with* aura (MA), and 18% (20% in women, 7% in men) had both MO and MA.

**FIGURE 2:**
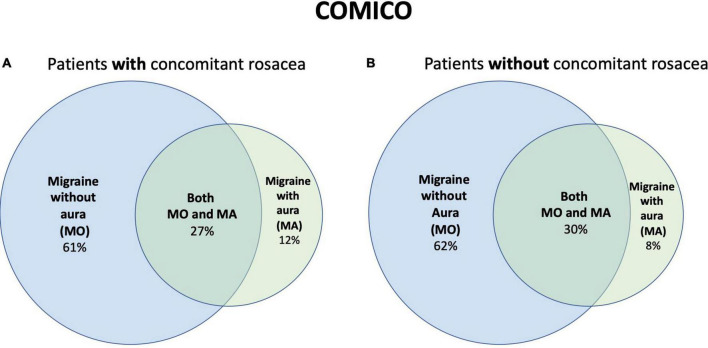
Distribution of migraine subtypes in COMICO in patients, **(A)** with concomitant rosacea; **(B)** without concomitant rosacea. COMICO, Copenhagen Migraine Cohort.

In COMICO, MO was present in 62% (61% in women, 70% in men), MA in 10% (10% in women, 15% in men), and both MO and MA in 28% (30% in women, 15% in men). There was no difference in prevalence of migraine subtypes in patients with/without rosacea in COMICO in adjusted analyses (*p* = 0.20) (Figure 2 and Supplementary Tables 2, 3).

### Migraine frequency

Of those fulfilling criteria for migraine in COROCO, 25% had not had an attack in the past year, 31% had less than 5 attacks, 25% had between 6 and 24 attacks in the past year, 4% had between 2 and 3 attacks per *month*, and 15% had more than 3 attacks per *month* (Supplementary Figure 2).

There was no difference in severity of migraine (episodic/chronic migraine) between patients with/without concomitant rosacea in COMICO (*p* = 0.96) (Supplementary Table 3).

### Rosacea

#### Prevalence and phenotype of rosacea

**FIGURE 3:**
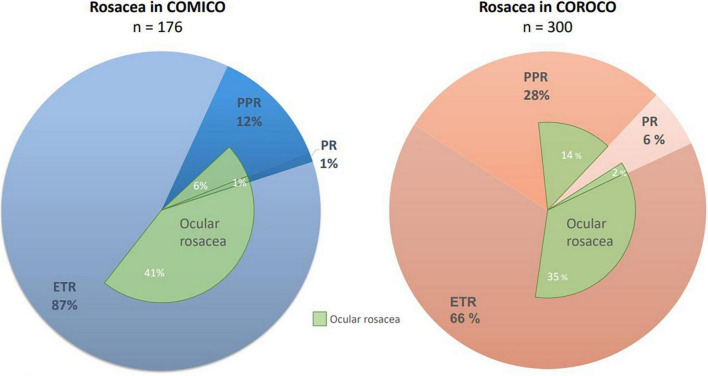
Distribution of rosacea subtypes in each cohort. ETR, erythematotelangiectatic rosacea; COROCO, Copenhagen Rosacea Cohort; COMICO, Copenhagen Migraine Cohort; PPR, papulopustular rosacea; OR, ocular rosacea; PR, phymatous rosacea.

In the migraine cohort, COMICO, the overall prevalence of rosacea was 65%. Rosacea prevalence exceeded 60% in women aged 18–54 years, with a drop after this age. For men, prevalence peaked above age 40 years (59%). There was an overlap of features and the most common rosacea feature in COMICO was *fixed centrofacial erythema in a characteristic pattern* (hereafter: *erythema*) (79%), followed by flushing (69%), dryness (59%), ocular symptoms (49%), telangiectasia (45%), burning (38%), papules/pustules (31%), edema (3%), and rhinophyma (1%). When looking at rosacea subtypes, 87% had erythematotelangiectatic rosacea (ETR), 12% had papulopustular rosacea (PPR), 1% had phymatous rosacea (PR), and 48% had ocular rosacea (Figure 3).

In COROCO, erythema was present in 98% (96% in patients *with* migraine, 99% in patients *without* migraine), flushing in 80% (72% in patients *with* migraine, 87% in patients *without* migraine), telangiectasia in 72% (72% in patients both *with* and *without* migraine), dryness was present in 62% (58% in patients *with* migraine, 66% in patients *without* migraine), papules/pustules were present in 60% (51% in patients with *migraine*, 67% in patients *without* migraine), burning in 60% (50% in patients *with* migraine, 68% in patients *without* migraine), ocular symptoms were present in 51% (39% in patients *with* migraine, 61% in patients *without* migraine), 10% (10% in patients *with* migraine, 9% in patients *without* migraine) experienced edema, and 6% (9% in patients *with* migraine, 4% in patients *without* migraine) had rhinophyma (Figure 3). In COROCO, 66% had ETR, 28% had PPR, 6% had PR, and 51% had ocular rosacea (Figure 3).

Concomitant migraine was significantly associated with flushing [OR = 2.6, 95% confidence interval (CI) = 1.4–4.7, *p* = 0.002], ocular symptoms (OR = 2.4, 95% CI = 1.5–3.9, *p* < 0.001), and burning (OR = 2.1, 95% CI = 1.3–3.4, *p* = 0.002), and *inversely* related with papules/pustules (OR = 0.5, 95% CI = 0.3–0.8, *p* = 0.006). Concomitant migraine in COROCO was *not* associated with erythema, rhinophyma, telangiectasia, edema, or dryness. For details, see Supplementary Table 5.

### Severity of rosacea

For severity of rosacea, CEA, IGA, and RASI were applied. In COMICO, 22% (23% in women, 21% in men) presented with almost clear rosacea, 55% (53% in women, 69% in men) had mild rosacea, 19% (21% in women, 5% in men) had moderate rosacea, and 4% (3% in women, 5% in men) had severe rosacea. Mean RASI was 10.3 (*SD* = 4.9) for patients *with* rosacea in COMICO.

In COROCO, mean RASI was 11.3 (*SD* = 5.6) in patients *with* migraine and 12.5 (*SD* = 6.2) in patients *without* migraine (*p* = 0.40). There was no difference in severity of rosacea in patients with/without concomitant migraine when looking at IGA or RASI, but when evaluating CEA we found that concomitant migraine was associated with a higher CEA (mean = 2.41, 95% CI, 2.25–2.57 for patients *with* concomitant migraine, and mean = 1.79, 95% CI, 1.59–1.99 for patients *without* concomitant migraine, *p* < 0.001).

For overview of rosacea severity in both cohorts, see Supplementary Table 4. Severity of migraine (chronic migraine) was not associated with a higher RASI score when adjusted for age, sex and smoking in COROCO (*p* = 0.09).

### Dermatology life quality index

In COROCO, mean dermatology life quality index (DLQI) was 3.0 (*SD* = 3.9) for those *without* migraine and 4.2 (*SD* = 4.8) for those *with* migraine (*p* = 0.04), although the difference was not significant when adjusted for age, sex, and smoking (*p* = 0.68). For patients *with* rosacea in COMICO, mean DLQI was 2.3 (*SD* = 3.1) compared with 1.1 (*SD*, 1.8) for patients *without* rosacea. The difference was significant when adjusting for age, sex, and smoking (*p* < 0.001). For overview of DLQI in cohorts, see Supplementary Table 4).

## Discussion

In these well-characterized hospital-based cohorts of patients with migraine or rosacea, we demonstrated a substantial overlap between the two diseases. More than 60% of the patients in the migraine cohort presented with rosacea features, and more than half of the patients in the rosacea cohort had migraine. Concomitant migraine was significantly associated with the rosacea features flushing, ocular symptoms and burning, and inversely related with papules/pustules. No association was found with erythema, rhinophyma, telangiectasia, edema, or dryness. No association was found between subtype of migraine and severity of rosacea in adjusted analyses.

### Migraine

#### Prevalence of migraine in patients with concomitant rosacea

Migraine is the leading cause of disability in under 50s leading to sick days and hugely affecting quality of life (2). In the rosacea cohort, COROCO, more than half of the patients had migraine, and 41% had had an attack in the past year. The lower 1-year prevalence of migraine in COROCO probably reflected the relatively high mean age in COROCO as migraine often improves after menopause (7). Although we did not include a control group, migraine in the background population has previously been extensively studied. Global prevalence of migraine (lifetime) is reported between 15 and 20% (3) and a recent cross-sectional study in *European* countries found a gender-adjusted *1-year* prevalence of 35%, with a peak between ages 30–40 years for men, and 20–60 years for women (26), which is consistent with the findings in our cohorts. Interestingly, a recent meta-analysis found an OR of 1.96 (95% CI = 1.41–2.72) for migraine in patients with rosacea compared with the background population (20). One study has also found the use of triptans to be associated with a slightly higher risk of incident rosacea in 53,927 females aged 60 years or older (OR 1.66, 95% CI = 1.30–2.10) (27). Authors of the latter study proposed that triptan use (vasoconstrictors) might *provoke* rosacea onset. However, we recently found experimentally induced rosacea features (erythema, flushing, and facial edema) to be *attenuated* by the anti- migraine drug sumatriptan (28). Further, a patient with severe attacks of rosacea flushing displaying migraine-like characteristics (general malaise, light-sensitivity, but without headache, lasting 2–3 days) was effectively treated with oral sumatriptan (29). This suggests that triptans may instead prove beneficial in rosacea, and that previous data could indicate a pathophysiological and/or genetic overlap between the two (20).

Interestingly, in COROCO, 40% of the patients with migraine were unaware of their diagnosis (unrecognized migraine). This implies that migraine is still largely underdiagnosed and undertreated. In comparison, in a Danish population-based twin study, unrecognized migraine was reported at 24% (22). A reason for the high proportion of unrecognized migraine could be that patients with rosacea in COROCO anecdotally reported their migraine headaches lower on numerical pain rating scales or with milder associated symptoms, although this information was not systematically collected.

The most common subtype of migraine in both cohorts was MO consistent with previous data (30). MA has previously been reported at 30% in Danish patients with migraine (30). The prevalence in our cohorts was slightly higher (38%) possibly due to a more thorough interview of symptoms. In COMICO, severity and subtype of migraine was unrelated to prevalence, severity, and subtype of rosacea.

### Rosacea

#### Prevalence and severity of rosacea in patients with concomitant migraine

A recent German study found a rosacea prevalence of 2.1% in both sexes. Prevalence increased with age, peaking at 5.7% for individuals aged 60–70 years (31). In the background population prevalence of rosacea ranged between 0.1 and 22.4% with an overall prevalence of 5.5% (1). In our migraine cohort, COMICO, almost two thirds of the patients had rosacea, and an additional 26% of those *without* rosacea reported to be frequent flushers. Flushing—in lack of other rosacea features—has been associated with a high prevalence of migraine (18). Interestingly, flushing is reported in 42% of patients with rosacea and 16% in healthy controls, and flushing has been suggested to be a sign of pre-rosacea (32, 33).

Of those fulfilling criteria for rosacea, 81% were unaware of this (unrecognized rosacea) consistent with previous population-based studies finding rosacea to be largely unrecognized and underdiagnosed (34, 35). Unrecognized rosacea was associated with mild features in 77% (women: 75%, men: 92%), moderate features in 19% (women: 21%, men: 0%) and severe features in 4% (women: 3%, men: 8%), indicating that unrecognized rosacea was not exclusively associated with low disease burden, but rather unawareness of rosacea as a diagnosis.

### Rosacea phenotype

Erythema was the most common feature in COMICO, followed by flushing, dryness, and ocular symptoms. This is consistent with a recent Danish population-based study which found concomitant migraine to be associated with the previously used ETR and ocular subtypes of rosacea (19). The patients who did not present with erythema either presented with phymatous changes, and/or by having at least two major rosacea features. Almost 1/3 of the patients were currently being treated (either locally or systemically) for their rosacea which may have also “masked” current features. A recent meta-analysis looked at the distribution of subtypes in patients with rosacea and found the most common subtype to be ETR (pooled proportion = 56.7%, 95% CI, 51.4–62.0%), followed by papulopustular rosacea (pooled proportion = 32.2%, 95% CI, 38.8–47.6%), ocular rosacea (pooled proportion = 11.1%, 95% CI, 6.7–16.3%) and phymatous rosacea (pooled proportion = 7.4%, 95% CI, 6.1–8.9%) (36). Migraine has been consistently associated with dry eye disease in population-based studies (37–42), although it has not been explored whether this dry eye disease might in fact be ocular rosacea. It is unclear what drives the correlation between migraine and ocular rosacea. High levels of proinflammatory markers as well as toll-like receptor 4 and human peptide LL-37 have been found in tears of patients with ocular rosacea (43) although it is not clear whether this indicates a connection with ocular rosacea or rather severity of inflammation, as we found concomitant migraine to be associated with higher CEA.

Presence of papules/pustules was inversely associated with concomitant migraine. Previous studies on the connection between migraine and rosacea have not been consistent in reporting subtypes/phenotypes, and only *one* small cross-sectional study has suggested a connection between migraine and papules/pustules (previously papulopustular rosacea) (44).

Rhinophyma was very uncommon in COMICO, although no significant differences were found between patients with/without concomitant migraine in COROCO. No studies have previously connected rhinophyma and migraine.

The pathophysiology behind both rosacea and migraine remains incompletely understood, but both have been associated with increases in neuropeptides such as calcitonin gene-related peptide and pituitary adenylate cyclase-activating polypeptide (20, 45–47). Interestingly, infusion of pituitary adenylate cyclase-activating polypeptide-38 can induce both migraine and rosacea in humans (28, 48). Monoclonal antibodies against pituitary adenylate cyclase-activating polypeptide are currently being developed, and monoclonal antibodies against calcitonin gene-related peptide were recently approved for preventive treatment of migraine (7). It could be speculated whether antibodies against these neuropeptides might also prove beneficial in rosacea. Indeed, monoclonal antibodies against calcitonin gene-related peptide are currently being tested in rosacea (Clinicaltrials.gov NCT04419259).

Furthermore, preventive treatment against migraine includes calcium channel blockers and riboflavin, which have been associated with an increased risk of incident rosacea/worsening of rosacea features (49) whereas beta blockers and sumatriptan, which are also used in the acute and preventive treatment of migraine may prove beneficial in rosacea (29, 49). Conversely, isotretinoin, which is used in rosacea treatment, is commonly associated with non-migraine headache (50), and it could be important to consider common side effects when choosing between treatment options in both rosacea and migraine.

### Strengths and limitations

There are several strengths in our cohorts. First, we conducted face-to-face interviews in a large population of patients—in terms of interview studies. A validated questionnaire was used for diagnosis of migraine. For rosacea, diagnosis was made during the interview and confirmed by clinical examination, supplemented with clinical photographs evaluated by experienced physicians.

Limitations include risk of recall bias as data were collected retrospectively, and the age and sex- difference between cohorts. The age difference could have affected prevalence of rosacea in the migraine cohort negatively, as rosacea usually affects individuals aged 30 years or above compared with a debut at 20–30 years for migraine (23, 51, 52). Although both rosacea and migraine are common in women, a recent review shows that the sex distribution in patients with rosacea is almost equal, whereas migraine remains most common in women (1, 7). Furthermore, we did not include any clinical tests for evaluating ocular symptoms, and these features may have been overestimated. For migraine, severity was only reported as episodic or chronic migraine, and it could be relevant to investigate headache pain severity in the future. Another limitation was the lack of a control group although prevalence of both diseases in the background population has previously been thoroughly investigated. Furthermore, patients were recruited from specialized tertiary clinics, investigating only those with a high burden of disease. In line with this, there was a risk of selection bias, as patients identifying with one of the diseases might be more prone to participate; however, the overall lack of research in rosacea seemed to be enough motivation for patients with rosacea, and the relatively short duration of the interviews (1 h) seemed short enough for patients with migraine to be willing to remain at the clinic following their out-patient visit.

## Conclusion

In conclusion, we found a strong co-occurrence of rosacea and migraine. Concomitant migraine was associated with flushing, ocular symptoms, and burning, and inversely associated with papules/pustules severity of rosacea. Concomitant migraine was associated with more severe erythema but with subtype or severity of migraine. The causal relationship between rosacea and migraine is unclear and would require follow up studies as well as genetic and experimental studies to uncover a possible pathophysiological link. Many patients were unaware of their concomitant disease with a risk of undertreatment or inappropriate treatment in patients, resulting in a high physical and psychological burden. Our findings highlight the need to consider comorbidities in these patients and the need for a multidisciplinary approach toward management of both diseases.

## Data availability statement

The original contributions presented in this study are included in the article/Supplementary material, further inquiries can be directed to the corresponding author/s.

## Ethics statement

The studies involving human participants were reviewed and approved by the Ethical Committee of the Capital Region of Denmark. The patients/participants provided their written informed consent to participate in this study.

## Author contributions

NW, CC, MA, JT, and AE: conceptualization, data curation, formal analysis, methodology, resources, supervision, and validation. NW and MA: funding acquisition and project administration. NW, DZ, A-SR, and HB: investigation. NW, CC, and MA: software and visualization. NW, MA, JT, and AE: writing—original draft preparation. All authors: writing—review and editing.

## Abbreviations

COMICO: Copenhagen Migraine Cohort
COROCO: Copenhagen Rosacea Cohort
IQR: interquartile range
MA: migraine with aura
MO: migraine without aura
OR: odds ratio
SD: standard deviation.
