# Supplementary material for: Clinical characteristics of combined rosacea and migraine
Source: Front Med (Lausanne). 2022 Oct 20;9:1026447. doi: 10.3389/fmed.2022.1026447 (PMC9635264; doi:10.3389/fmed.2022.1026447)
Supplement: Supplementary file 1 [file Data_Sheet_1.PDF]

Supplement 1:

Semi-structured interview for diagnosing headache and migraine. Adapted from a validated interview from the Danish Headache Center (last updated November 18, 2012) for the purpose of interviewing patients without a diagnosis of migraine.

Semi-Structured Migraine and Headache Interview

0. Headache

0.1 Have you been diagnosed with migraine

□ Yes □ No

0.1.1 If yes, did anything happen in relation to debut of migraine?

□ Yes □ No

0.1.1.1 If yes – what happened

□ Menarche
□ Head trauma / Concussion
□ Other _______________

0.1.2 If NO:

0.1.2.1 Do you experience regular headaches?

0.1.2.1.1 If yes, how often (days per month) ___

0.1.2.1.2 Is the headache related to anything in particular

□ Yes □ No

0.1.2.1.3 If yes, what? ____________________

<u>For all patients:</u>

Do you ever experience headaches that are:

	Yes	No
a. Unilateral	□ 1	□ 2
b. Pulsating	□ 1	□ 2
c. Moderate/severe intensity	□ 1	□ 2
d. Aggravation by physical activity	□ 1	□ 2
e. Nausea	□ 1	□ 2
f. Vomiting	□ 1	□ 2
g. Photofobia	□ 1	□ 2
h. Phonofobia	□ 1	□ 2
i. Osmophoabia	□ 1	□ 2

Duration of the headache without medication:

< ½ h □ 1
½ - 4 h □ 2
5 h – 23 h □ 3
1 - 3 days □ 4
4 – 7 days □ 5
>7 days □ 6

1. <u>MIGRAINE WITH AURA (MA)</u>

a. Do you have migraine with aura? **□ 1** **□ 2**

**1.1 Visual aura**	Yes	No
a. Are there visual disturbances?	□ 1	□ 2
b. Unilateral	□ 1	□ 2
c. Gradually progressing	□ 1	□ 2
d. Scotoma	□ 1	□ 2
e. Zig-zag lines (fortification)	□ 1	□ 2
f. Flickering	□ 1	□ 2
g. Preserved central vision	□ 1	□ 2
h. Duration of gradual development	______min	
j. Duration of visual aura	_______min	

**1.2 Sensory aura**	Yes	No
a. Are there sensory disturbances?	□ 1	□ 2
b. Unilateral	□ 1	□ 2
c. Gradually progressing	□ 1	□ 2
Do the sensory disturbances involve:		
d. The face	□ 1	□ 2
e. The tongue	□ 1	□ 2
f. The hand	□ 1	□ 2
g. The arm	□ 1	□ 2
h. The foot	□ 1	□ 2
i. The leg	□ 1	□ 2
j. The body	□ 1	□ 2
k. Duration of gradual development	______min	
l. Duration of visual aura	_______min	

**1.3 Motor aura**	Yes	No
a. Are there motor disturbances?	□ 1	□ 2
b. Unilateral	□ 1	□ 2
c. Gradually progressing	□ 1	□ 2
Do the motor disturbances involve:		
d. The face	□ 1	□ 2
e. The tongue	□ 1	□ 2
f. The hand	□ 1	□ 2
g. The arm	□ 1	□ 2
h. The foot	□ 1	□ 2
i. The leg	□ 1	□ 2
j. The body		
k. Duration of gradual development	______min	
l. Duration of visual aura	_______min	

**1.4 Aphasia/ Speech disturbances**	Yes	No
a. Are there speech disturbances?	□ 1	□ 2
Are the speech impairments due to:		
b. Problems articulating speech	□ 1	□ 2
c. Problems finding the right words	□ 1	□ 2
d. Problems understanding what people say	□ 1	□ 2
e. Problematic for other people to understand your speech	□ 1	□ 2
f. Duration of speech/aphasic disturbances _______________min		

1.5 Basilar-type aura Yes No

	Yes	No
a. Are there basilar/occipital symptoms?	□ 1	□ 2
Are there:		
b. Bilateral pareses/parestesias	□ 1	□ 2
c. Bilateral visual symptoms	□ 1	□ 2
d. Dysarthria	□ 1	□ 2
e. Vertigo	□ 1	□ 2
f. Diplopia	□ 1	□ 2
g. Tinnitus	□ 1	□ 2
h. Hypacusia	□ 1	□ 2
i. Decreased level of consciousness	□ 1	□ 2
j. Ataxia	□ 1	□ 2

1.6 Succession of aura symptoms

a. If more than 1 aura type, is the succession of the auras:

Successive □ 1
Simultaneously □ 2
Not applicable (NA) □ 3

1.7 Aura with headache Yes No

a. Do you have aura with headache □ 1 □ 2

b. Does the onset of the headache typically come:

Before the aura □ 1
After the aura □ 2
Simultaneously with the aura □ 3

c. How long time before/after the aura_____min

1.8 Aura without headache Yes No

a. Do you have aura without headache □ 1 □ 2

1.9 **Migraine with aura over time**

a. Age at onset_____years
b. Date of last attack_____
c. No. of attacks within last year:

0 □ 1
1-5 □ 2
6-12 □ 3
13-24 □ 4
25-36 □ 5
>36 □ 6

d. No. of lifetime attacks:

1 □ 1
2-4 □ 2
5-9 □ 3
10-49 □ 4
50-100 □ 5
>100 □ 6

2. MIGRAINE WITHOUT AURA (MO)

a. Do you have migraine without aura? **Yes □ 1** **No □ 2**

2.1 Migraine without aura over time

a. Age at onset_____years
b. Date of last attack_____
c. No. of attacks within last year:

0 □ 1
1-5 □ 2
6-12 □ 3
13-24 □ 4
25-36 □ 5
>36 □ 6

d. No. of lifetime attacks:

1 □ 1
2-4 □ 2
5-9 □ 3
10-49 □ 4
50-100 □ 5
>100 □ 6

3. Migraine triggers

	Yes	No	NA
**a.** Are there factors that can trigger a migraine attack?	□ 1	□ 2	□ 3

	**MO**	**MA**	**MA+MO**
**b.** What type of migraine?	□ 1	□ 2	□ 3

3.1. Can these factors trigger a migraine attack:

	Yes	No
a. Physical activity	□ 1	□ 2
b. Light	□ 1	□ 2
c. Stress	□ 1	□ 2
d. Menstruation	□ 1	□ 2
e. Alcohol	□ 1	□ 2
f. Strong smells	□ 1	□ 2
g. Lack of/too much sleep	□ 1	□ 2

h. Other factors:________________________

4. Chronic migraine (MA+MO)

During the past 3 successive months, have you had:

	Yes	No
a. Headache at least 15 days a month	□ 1	□ 2
b. Migraine at least 8 days a month	□ 1	□ 2

5. Tension-type headache

	Yes	No
**Do you have tension-type headaches**	□ 1	□ 2

5.1 Headache characteristics

	Yes	No
a. Bilateral	□ 1	□ 2
b. Pressing	□ 1	□ 2
c. Mild/moderate intensity	□ 1	□ 2
d. Aggravation by physical activity	□ 1	□ 2
e. Nausea	□ 1	□ 2
f. Vomiting	□ 1	□ 2
g. Photofobia	□ 1	□ 2
h. Phonofobia	□ 1	□ 2

5.2 Duration of headache

< ½ h	□ 1
½ - 4 h	□ 2
5 h – 23 h	□ 3
1 - 3 days	□ 4
4 – 7 days	□ 5
>7 days	□ 6

5.3 Tension-type headache over time

a. Headache days within last year:

0	□ 1
1-7	□ 2
8-14	□ 3
15-30	□ 4
31-179	□ 5
≥180	□ 6

b. No. of tension-type headache days during the three last months:___________ days

c. If ≥45 headache days, are the days evenly spaced out

Yes	No
□ 1	□ 2

6. MIGRAINE TREATMENT (MA+ MO)

6.1 Treatment of migraine attacks

	Yes	No	NA
a. Triptans are efficient	□ 1	□ 2	□ 3
b. Regular painkillers (NSAID, Paracetamol etc.) are efficient	□ 1	□ 2	□ 3
c. Ergotamine drugs are efficient	□ 1	□ 2	□ 3

d. Other drug(s)________________________________

6.2 Use of medication

a. No. of days of triptan-use per month ________________

b. No. of days of regular painkiller-use per month ________

6.3 Prophylactic treatment of migraine

	Yes	No	NA
a. Beta-blockers are efficient	□ 1	□ 2	□ 3
b. Ca^{2+}-antagonists are efficient	□ 1	□ 2	□ 3
c. Angiotensin II receptor blockers are efficient	□ 1	□ 2	□ 3
d. ACE-inhibotors are efficient	□ 1	□ 2	□ 3
e. Anti-epilepsy drugs are efficient	□ 1	□ 2	□ 3
f. Antidepressive medication (mirtazapine) is efficient	□ 1	□ 2	□ 3
g. Hormone treatment is efficient	□ 1	□ 2	□ 3

h. Other drug(s) ________________________________

	Yes	No
g. Are you currently receiving prophylactic treatment(s) for migraine	□ 1	□ 2

8. SECONDARY HEADACHES?

Yes	No
□ 1	□ 2

If yes, specify:_______________________________

11. Migraine within the family

	Yes	No
a. Mother has/had migraine	□ 1	□ 2
b. Father has/had migraine	□ 1	□ 2
d. Siblings have/had migraine	□ 1	□ 2
e. Children have/had migraine	□ 1	□ 2

Interview conducted by:________________________
