# Supplementary material for: Clinical characteristics of combined rosacea and migraine
Source: Front Med (Lausanne). 2022 Oct 20;9:1026447. doi: 10.3389/fmed.2022.1026447 (PMC9635264; doi:10.3389/fmed.2022.1026447)
Supplement: Supplementary file 2 [file Data_Sheet_2.PDF]

Supplement 2. Semi-structured interview developed at the department of Dermatology in Gentofte, Denmark by authors NW, JT and AE. The purpose of the interviews is to uncover rosacea features, previous treatments for rosacea, and comorbidities in the patient and in 1st and 2nd degree relatives. The interview also includes sleeping habits, smoking, alcohol, BMI, dermatology life quality index (DLQI) and rosacea clinical scorecard.

1. Rosacea

1.1 Has a doctor ever told you that you have rosacea? (one answer)

- ☐ No – and I do not have rosacea
- ☐ Yes, I am certain I have rosacea, but a doctor has never told me.
- ☐ Yes – a doctor who is not a dermatologist (e.g. GP)
- ☐ Yes – a dermatologist

If yes to one of the above, go to question. 1.2. If no, move on to question 3

1.2 Which symptom(s) of rosacea did you first notice? (multiple answers)

- ☐ Redness of particularly cheeks and/or the chest, which did not want to go away
- ☐ Flushing attacks (sudden warmth/burning sensations and redness which lasts a few minutes – half an hour)
- ☐ Persistent (> 1 hour) attacks of flushing
- ☐ Telangiectasias in the face (cheeks, nose, chin or eyelids)
- ☐ Symptoms from the eyes
- ☐ Recurrent formation of pimples in the face
- ☐ Change of the nose's look or size
- ☐ Other? ____________________________________

1.2.1 At what age did you experience the first symptom(s) of rosacea? Age ☐☐ years

1.2.2 How much time passed from your first symptom(s) of rosacea until a doctor diagnosed you with rosacea?
Year ☐☐ Months ☐☐

1.3 Has any of the following symptoms appeared started appearing since you noticed the first symptom(s) of rosacea? (multiple answers)

- ☐ Redness of particularly cheeks and/or the chest, which did not want to go away
- ☐ Flushing attacks (sudden warmth/burning sensations and redness which lasts a few minutes – half an hour)
- ☐ Persistent (> 1 hour) attacks of flushing
- ☐ Telangiectasias in the face (cheeks, nose, chin or eyelids)
- ☐ Symptoms from the eyes
- ☐ Recurrent formation of pimples in the face
- ☐ Change of the nose's look or size
- ☐ Other? ____________________________________

1.4 Do you still have symptoms of Rosacea? (one answer)

☐ No ☐ Improvement ☐ Worsening ☐ Unchanged symptoms

Describe:

2. ROSACEA TREATMENTS

2.1 Have you ever been treatment for rosacea? (one aswer)

☐ **No**, never (move on to question 3)
☐ Yes, but I am no longer in treatment
☐ Yes, I still receive treatment

2.2 How long did/have you receive(d) treatment for rosacea? (cumulated time)

☐ Less than 3 months ☐ 3 months – 1 year ☐ More than 1 year – how long (years)___________________

2.3 If no longer in treatment for rosacea – why did you stop treatment? (one answer)

☐ My symptoms improved / disappeared after treatment
☐ There was no effect of the treatment on my symptoms
☐ My symptoms worsened due to treatment
☐ I got side effects from the treatment
☐ I do not wish to be on daily medication

2.4 Which type of treatment(s) have you received? (multiple answers)

☐ Creme/gel/ointment ☐ Pills ☐ Laser treatment

2.5 Which drug(s) have you tried, and did it/they have any effect? (multiple answers)	Yes	No	Do not know
☐ Mirvaso (brimonidine tartrate) creme/gel	☐	☐	☐
☐ Finacea (azelaic acid) creme/gel	☐	☐	☐
☐ Metronidazole / metrocrem / rozex / robaz creme/gel	☐	☐	☐
☐ Oracea (doxycycline) tablet	☐	☐	☐
☐ Soolantra (ivermectin) creme	☐	☐	☐
☐ Tetracycline	☐	☐	☐
☐ Erythromycin (macrolide) tablet	☐	☐	☐
☐ Accutin / Isotretinoin tablet	☐	☐	☐
☐ Other: ______________________________________			

2.6 Which symptom(s) did the treatment influence? (multiple answers)

☐ Papules and pustules (impurities/pimples)
☐ Unwanted redness of the face
☐ Telangiectasias in the face
☐ Eye symptoms
☐ Nose Changes
☐ Other: _______________________________

Other comments to treatment:

3. FLUSHING + OTHER SYMPTOMS

REDNESS/ SENSITIVE SKIN

3.1 Are any areas of your face often pink or red? ☐ No ☐ Yes

3.2 Is your face often pink or red compared with other people? ☐ No ☐ Yes

3.3 Is your face often pink or red compared to other body areas (e.g. abdomen, upper arms) ☐ No ☐ Yes

3.4 Have others previously mentioned that your face was pink or red? ☐ No ☐ Yes

3.5 Do you experience that coldness, heat or direct sunlight can provoke a facial burning/stinging sensation after only short exposure?

☐ No ☐ Rarely ☐ In periods (e.g. winter) ☐ Monthly ☐ Weekly ☐ Daily

3.6 Do you experience dry/scaly skin in central areas of your face, e.g. where you usually experience redness?

☐ No ☐ Rarely ☐ In periods (e.g. winter) ☐ Monthly ☐ Weekly ☐ Daily

3.7 Is you skin sensitive, i.e. blushes easily and/or gets tight/dry easily?

☐ No ☐ Rarely ☐ In periods (e.g. winter) ☐ Monthly ☐ Weekly ☐ Daily

TELANGIECTASIAS

3.8 Do you have telangiectasias in the face (e.g. around the nose or center of the cheeks)? ☐ No ☐ Yes

3.8.1 If yes, where are the telangiectasias located?

☐ on top of the nose ☐ sides of the nose ☐ cheeks ☐ chin ☐ eyelids ☐ other:___________________________

FLUSHING

3.9 Have you experienced flushing in the ***past year***?

☐ **No,** not at all ☐ Yes, a few times (less than 12 times) ☐ Yes, periodically ☐ Monthly ☐ Weekly ☐ Daily

3.9.1 In your experience, was the start of flushing related to something?

☐ **no** ☐ menopause (hot flushes) ☐ high/low metabolism ☐ medication ☐ other_________________________

3.9.2 If yes to flushing, in which areas of the skin do you experience flushing?

☐ forehead ☐ center of the cheeks ☐ nose ☐ ears ☐ chin ☐ neck ☐ chest

3.9.3 How long does a (severe) flushing last? (describe any other symptoms)

____

3.10 As a ***child or teenager***, did you experience that your face would easily become red (e.g. when you were nervous/shy or exercised)

☐ No, never
☐ Yes, I have experienced it a couple of times (few times a year or less)
☐ It happened occasionally/frequently
☐ I would always blush when I got embarrassed
☐ I experienced it daily and sometimes without a trigger

3.10.1 How old were you the first time you experienced flushing? Age ☐☐ years

3.11 Can any of the following give you a sudden sensation of warmth (flushing) (multiple answers)

No Yes
- ☐ ☐ Alcohol
- ☐ ☐ Hot food or drinks
- ☐ ☐ Spicy food
- ☐ ☐ Sunlight
- ☐ ☐ Hot and humid surrounds e.g. sauna or hot bath etc.
- ☐ ☐ Physical activity (e.g. sport)
- ☐ ☐ Psychological stress or emotional revolt (e.g. holding a speech in front of a large audience)
- ☐ ☐ Other: ____
- ☐ None of the above

3.12 **Do you experience having thickened skin on your nose** ☐ Yes ☐ No

4. ACNE

4.1 Have you experienced frequently having impure skin/pimples in the face after becoming an adult (above 25 years of age)

- ☐ No (Go to question 5)
- ☐ No, but I had acne when I was younger
- ☐ Yes, I have previously experienced pimples, which occurred after I became an adult, but I do not anymore
- ☐ Yes, and I still frequently experience having pimples

4.2 If yes, do they occur in relation to anything special?

☐ No ☐ Periods ☐ Alcohol ☐ Other ____

4.3 Where are these impurities/pimples typically located when you have them? (multiple answers)

☐ Forehead ☐ Cheeks ☐ Nose ☐ Chin ☐ Chest ☐ Back ☐ Shoulders ☐ Other ____

5. EYE SYMPTOMS

5.1 Do you **frequently** experience

No Yes
- ☐ ☐ red/bloodshot eyes
- ☐ ☐ watery/runny eyes
- ☐ ☐ foreign body sensation of the eyes
- ☐ ☐ stinging sensation in eye/eyes
- ☐ ☐ itching sensation in eye/eyes
- ☐ ☐ small, fine scales around eyelid margins
- ☐ ☐ thickened sensation of eyelid(s), which can be sore or red
- ☐ ☐ feeling the need to close eyes in the evening, in air-conditioned spaces, during flights etc.

5.2 If yes to any of the above, have you ever visited an ophthalmologist due to these symptoms? ☐ No ☐ Yes

5.3 Have you had the need to use viscous/watery eyedrops (artificial tears) for longer/shorter periods of time? ☐ No ☐ Yes

6. TREATMENT WITH CORTICOSTEROIDS/ADRENOCORTICAL HORMONE

6.1 Have you ever been treated with corticosteroids (also called adrenocortical hormone or prednisolone)?

☐ No, never (move on to question 7) ☐ Yes – creme/ointment ☐ Yes – pills ☐ Yes – syringe

6.2 Have you ever been treated with corticosteroids/adrenocortical hormone?

☐ No, never ☐ Yes, a short period of time (less than 1 month cumulated) ☐ Yes, a longer period (1-12 months cumulated)

☐ Yes, a long period (>12 months cumulated)

6.2.1 If yes, at what age were you when you were first treated with corticosteroids in the face? Age ☐☐ years

7. OTHER DISEASES AND TREATMENT

7.1 Has a doctor ever told you or someone in your family that you/they had any of the following diseases? (Only biologically related family members, i.e. not stepsister or stepparents)

SKIN

Rosacea
☐ no one ☐ me ☐ parent(s) ☐ sibling(s) ☐child(ren) ☐ grandparent(s) ☐grandchild(ren) ☐parent(s) brother/sister(s) ☐niece/nephew(s)

Acne
☐ no one ☐ me ☐ parent(s) ☐ sibling(s) ☐child(ren) ☐ grandparent(s) ☐grandchild(ren) ☐parent(s) brother/sister(s) ☐niece/nephew(s)

Seborrheic dermatitis
☐ no one ☐ me ☐ parent(s) ☐ sibling(s) ☐child(ren) ☐ grandparent(s) ☐grandchild(ren) ☐parent(s) brother/sister(s) ☐niece/nephew(s)

Psoriasis
☐ no one ☐ me ☐ parent(s) ☐ sibling(s) ☐child(ren) ☐ grandparent(s) ☐grandchild(ren) ☐parent(s) brother/sister(s) ☐niece/nephew(s)

Atopic dermatitis
☐ no one ☐ me ☐ parent(s) ☐ sibling(s) ☐child(ren) ☐ grandparent(s) ☐grandchild(ren) ☐parent(s) brother/sister(s) ☐niece/nephew(s)

Non-melanoma skin cancer
☐ no one ☐ me ☐ parent(s) ☐ sibling(s) ☐child(ren) ☐ grandparent(s) ☐grandchild(ren) ☐parent(s) brother/sister(s) ☐niece/nephew(s)

Malignant melanoma
☐ no one ☐ me ☐ parent(s) ☐ sibling(s) ☐child(ren) ☐ grandparent(s) ☐grandchild(ren) ☐parent(s) brother/sister(s) ☐niece/nephew(s)

Urticaria (hives)
☐ no one ☐ me ☐ parent(s) ☐ sibling(s) ☐child(ren) ☐ grandparent(s) ☐grandchild(ren) ☐parent(s) brother/sister(s) ☐niece/nephew(s)

Any other skin disorder
☐ no one ☐ me ☐ parent(s) ☐ sibling(s) ☐child(ren) ☐ grandparent(s) ☐grandchild(ren) ☐parent(s) brother/sister(s) ☐niece/nephew(s)

Please describe: ____

PSYCHIATRIC

Anxiety
☐ no one ☐ me ☐ parent(s) ☐ sibling(s) ☐child(ren) ☐ grandparent(s) ☐grandchild(ren) ☐parent(s) brother/sister(s) ☐niece/nephew(s)

If ‘me’, have you ever been treated for anxiety? ☐ No, never ☐ Yes, and I am still in treatment ☐ Yes, but I am no longer in treatment

Depression
☐ no one ☐ me ☐ parent(s) ☐ sibling(s) ☐child(ren) ☐ grandparent(s) ☐grandchild(ren) ☐parent(s) brother/sister(s) ☐niece/nephew(s)

If ‘me’, have you ever been treated for depression? ☐ No, never ☐ Yes, and I am still in treatment ☐ Yes, but I am no longer in treatment

Any other phychiatric disorder
☐ no one ☐ me ☐ parent(s) ☐ sibling(s) ☐child(ren) ☐ grandparent(s) ☐grandchild(ren) ☐parent(s) brother/sister(s) ☐niece/nephew(s)

Please describe: ____

STOMACH AND GUT

Heartburn/reflux
☐ no one ☐ me ☐ parent(s) ☐ sibling(s) ☐child(ren) ☐ grandparent(s) ☐grandchild(ren) ☐parent(s) brother/sister(s) ☐niece/nephew(s)

Inflammatory bowel disease (Crohn’s disease/Ulcerative colitis)
☐ no one ☐ me ☐ parent(s) ☐ sibling(s) ☐child(ren) ☐ grandparent(s) ☐grandchild(ren) ☐parent(s) brother/sister(s) ☐niece/nephew(s)

Gluten intolerance/coeliac disease
☐ no one ☐ me ☐ parent(s) ☐ sibling(s) ☐child(ren) ☐ grandparent(s) ☐grandchild(ren) ☐parent(s) brother/sister(s) ☐niece/nephew(s)

Do you frequently experience discomfort/bloating and changing bowel habits? (Irritated bowel syndrome)
☐ No ☐ Rarely (few times a year) ☐ Monthly ☐ Weekly ☐ Daily

OTHER DISEASES

Type 1 diabetes
☐ no one ☐ me ☐ parent(s) ☐ sibling(s) ☐child(ren) ☐ grandparent(s) ☐grandchild(ren) ☐parent(s) brother/sister(s) ☐niece/nephew(s)

Type 2 diabetes
☐ no one ☐ me ☐ parent(s) ☐ sibling(s) ☐child(ren) ☐ grandparent(s) ☐grandchild(ren) ☐parent(s) brother/sister(s) ☐niece/nephew(s)

Sjogren’s syndrome
☐ no one ☐ me ☐ parent(s) ☐ sibling(s) ☐child(ren) ☐ grandparent(s) ☐grandchild(ren) ☐parent(s) brother/sister(s) ☐niece/nephew(s)

Metabolic disease
☐ no one ☐ me ☐ parent(s) ☐ sibling(s) ☐child(ren) ☐ grandparent(s) ☐grandchild(ren) ☐parent(s) brother/sister(s) ☐niece/nephew(s)

High cholesterol
☐ no one ☐ me ☐ parent(s) ☐ sibling(s) ☐child(ren) ☐ grandparent(s) ☐grandchild(ren) ☐parent(s) brother/sister(s) ☐niece/nephew(s)

Hypertension (high blood pressure)
☐ no one ☐ me ☐ parent(s) ☐ sibling(s) ☐child(ren) ☐ grandparent(s) ☐grandchild(ren) ☐parent(s) brother/sister(s) ☐niece/nephew(s)

If ‘me’, are you taking any treatment for hypertension? ☐ No ☐ Yes, pills, If yes, describe:____________________________

Raynaud’s phenomenon
☐ no one ☐ me ☐ parent(s) ☐ sibling(s) ☐child(ren) ☐ grandparent(s) ☐grandchild(ren) ☐parent(s) brother/sister(s) ☐niece/nephew(s)

AIRWAYS

COPD (chronic obstructive pulmonary disease)

☐ no one ☐ me ☐ parent(s) ☐ sibling(s) ☐child(ren) ☐ grandparent(s) ☐grandchild(ren) ☐parent(s) brother/sister(s) ☐niece/nephew(s)

Asthma

☐ no one ☐ me ☐ parent(s) ☐ sibling(s) ☐child(ren) ☐ grandparent(s) ☐grandchild(ren) ☐parent(s) brother/sister(s) ☐niece/nephew(s)

Hay fever

☐ no one ☐ me ☐ parent(s) ☐ sibling(s) ☐child(ren) ☐ grandparent(s) ☐grandchild(ren) ☐parent(s) brother/sister(s) ☐niece/nephew(s)

NEUROLOGICAL

Parkinson's disease

☐ no one ☐ me ☐ parent(s) ☐ sibling(s) ☐child(ren) ☐ grandparent(s) ☐grandchild(ren) ☐parent(s) brother/sister(s) ☐niece/nephew(s)

Alzheimer's disease

☐ no one ☐ me ☐ parent(s) ☐ sibling(s) ☐child(ren) ☐ grandparent(s) ☐grandchild(ren) ☐parent(s) brother/sister(s) ☐niece/nephew(s)

7.2 Do you often experience having cold nose and/or hands? ☐ No ☐ Yes, nose ☐ Yes, hands

7.3 Have you been diagnosed with/treated for any other diseases? ☐ No ☐ Yes, please describe: ______________________________

8. SLEEP

8.1 How often do you find it difficult to fall asleep?

☐ Once a month or less (never) ☐ 2-4 times a month ☐ one to several times a week ☐ daily

8.2 How often do you wake up earlier than what you intended (without being woken by an alarm or other noise)?

☐ Once a month or less (never). ☐ 2-4 times a month ☐ one to several times a week ☐ daily

9. SMOKING

9.1 Do you smoke? (one answer)

☐ No, I have never smoked ☐ No, but I have previously smoked ☐ Yes, occasionally (less than 1 cigarette per day). ☐ Yes, daily

ANSWERS FROM DAILY SMOKERS

9.2 How many cigarettes do you smoke on average? (daily number of cigarettes)

Number of cigarettes ☐☐ Other, describe: ________________________________

ANSWERS FROM OCCASIONAL SMOKERS

9.3 How many cigarettes do you smoke on average a week? (weekly number of cigarettes)

Number of cigarettes ☐☐ Other, describe: ________________________________

ANSWERS FROM OCCASIONAL AND FORMER SMOKERS

9.4 **Have you previously smoked every day?** ☐ yes ☐ no

9.5 If yes, how much did you smoke on average a day?

Number of cigarettes ☐☐ Other, describe: ________________________________

9.6 When did you stop smoking daily? (which year) ☐☐☐☐

ANSWER FROM ALL SMOKERS

9.7 How old were you when you started smoking? (age in years) ☐☐ years

10. ALCOHOL

10.1 Have you been drinking alcohol in the past year? ☐ No ☐ Yes

10.2 How much was you average weekly intake during the past 12 months? (Write '0' if none)☐☐ drinks per week

11. HEIGHT AND WEIGHT

11.1 What is your current height (without shoes)? ______________________________ cm

11.2 What is your current weight without clothes and shoes? ______________________kg

12. DERMATOLOGY LIFE QUALITY INDEX (DLQI)

12.1 Within the past week to what extent has your skin been itching, sore, hurting or stinging?
☐ Extremely ☐ Very ☐ A bit ☐ Not at all

12.2 Within the past week to what extent have you been embarrassed or shy because of your skin?
☐ Extremely ☐ Very ☐ A bit ☐ Not at all

12.3 Within the past week to what extent has your skin bothered you in terms of shopping or taking care of your house or back yard?
☐ Extremely ☐ Very ☐ A bit ☐ Not at all ☐ Not relevant

12.4 Within the past week to what extent has your skin affected the way you dress?
☐ Extremely ☐ Very ☐ A bit ☐ Not at all ☐ Not relevant

12.5 Within the past week to what extent has you skin affected your social activities or leisure activities?
☐ Extremely ☐ Very ☐ A bit ☐ Not at all ☐ Not relevant

12.6 Within the past week to what extent has you skin complicated your opportunities of exercise?
☐ Extremely ☐ Very ☐ A bit ☐ Not at all ☐ Not relevant

12.7 Within the past week has your skin prevented you from working or studying?
☐ Yes ☐ No ☐ Not relevant

If "No", within the past week has your skin been a problem for you at work or during studies?
☐ Extremely ☐ Very ☐ A bit ☐ Not at all

12.8 Within the past week to what extent has your skin caused problems in relation to your partner, close friends or relatives?
☐ Extremely ☐ Very ☐ A bit ☐ Not at all ☐ Not relevant

12.9 Within the past week to what extent has your skin caused sexual problems?
☐ Extremely ☐ Very ☐ A bit ☐ Not at all ☐ Not relevant

12.10 Within the past week, has treatment of your skin caused problems, e.g. by making your home messy or dirty, or by being time consuming?
☐ Extremely ☐ Very ☐ A bit ☐ Not at all ☐ Not relevant
