# Supplementary material for: Clinical characteristics of combined rosacea and migraine
Source: Front Med (Lausanne). 2022 Oct 20;9:1026447. doi: 10.3389/fmed.2022.1026447 (PMC9635264; doi:10.3389/fmed.2022.1026447)
Supplement: Supplementary file 3 [file Table_1.pdf]

Supplementary Table 1. Baseline characteristics for each cohort.

	COROCO			COMICO		
	**All** n=300	**Without Migraine** n = 137	**With Migraine** n=163	**All** n=304	**Without Rosacea** n=108	**With Rosacea** n=196
**Age, years, mean (SD)**	50.2 (12.9)	54.1 (12.0)	46.9 (12.7)	40.8 (12.9)	41.8 (13.7)	40.2 (12.4)
**Age at onset, years, mean (SD)**						
Of Rosacea	26.6 (13.4)	40.1 (15.0)	33.9 (13.6)	36.7 (14.6)	-	36.7 (14.6)
Of Migraine	24.3 (16.3)	-	24.3 (16.3)	25.3 (17.3)	26.2 (17.3)	24 (17.3)
**Sex, n (%)**						
Females	203 (67.7)	69 (50.4)	134 (82.2)	269 (88.5)	92 (85.2)	177 (90.3)
Males	97 (32.3)	68 (49.6)	29 (17.8)	35 (11.5)	16 (14.8)	19 (9.7)
**First-degree relative, n (%)**						
With Rosacea	124 (41.3)	52 (38.0)	72 (44.2)	45 (14.8)	12 (11.1)	33 (16.8)
With Migraine	117 (39.0)	45 (32.8)	73 (44.8)	193 (63.5)	61 (56.5)	130 (66.3)
**BMI, mean (SD), kg/m2**	26.4 (4.9)	26.2 (4.3)	26.5 (5.4)	25.5 (5.3)	24.6 (5.0)	26.0 (5.5)
**BMI groups, n (%)**						
BMI < 20	17 (5.7)	6 (4.4)	11 (6.7)	29 (9.5)	14 (13.0)	15 (7.7)
BMI 20-24	108 (36.0)	48 (35.0)	60 (36.8)	133 (43.8)	53 (49.1)	80 (40.8)
BMI 25-29	122 (40.7)	63 (46.0)	59 (36.2)	89 (29.3)	26 (24.1)	63 (32.1)
BMI 30 - 34	33 (11)	14 (10.2)	19 (11.7)	32 (10.5)	10 (9.3)	22 (11.2)
BMI 35-40	16 (5.3)	5 (3.6)	11 (6.7)	17 (5.6)	4 (3.7)	13 (6.6)
BMI > 40	4 (1.3)	1 (0.7)	3 (1.8)	4 (1.3)	1 (0.9)	3 (1.5)
**Smoking, n (%)**						
Current	39 (13.0)	19 (13.9)	20 (12.3)	52 (17.1)	14 (13.0)	38 (19.4)
Former	111 (36.6)	60 (43.8)	51 (31.3)	79 (26.0)	28 (25.9)	51 (26.0)
Never	151 (49.8)	58 (42.3)	92 (56.4)	173 (56.9)	66 (61.1)	107 (54.6)

Abbreviations: COMICO, Copenhagen Migraine Cohort; COROCO, Copenhagen Rosacea Cohort; n, number of patients; SD, standard deviation.
