# Supplementary material for: Clinical characteristics of combined rosacea and migraine
Source: Front Med (Lausanne). 2022 Oct 20;9:1026447. doi: 10.3389/fmed.2022.1026447 (PMC9635264; doi:10.3389/fmed.2022.1026447)
Supplement: Supplementary file 4 [file Table_2.pdf]

Supplementary Table 2. Subtype and severity of migraine in each cohort.

		**Migraine in COROCO**			**Migraine in COMICO**		
		**All** n = 163	**Women** n = 134	**Men** n = 29	**All** n = 304	**Women** n = 269	**Men** n = 35
**Subtype**	Migraine with aura, n (%)	32 (19.6)	22 (16.4)	10 (34.5)	31 (10.2)	26 (9.7)	5 (14.3)
	Migraine without aura, n (%)	102 (62.6)	85 (63.4)	17 (58.6)	188 (61.8)	163 (60.6)	25 (71.4)
	Both migraine with and without aura, n (%)	29 (17.8)	27 (20.1)	2 (6.9)	85 (28.0)	80 (29.7)	5 (14.3)
**Severity**	Chronic migraine, n (%)	12 (7.4)	11 (8.2)	1 (3.4)	160 (52.6)	141 (52.4)	19 (54.3)

Abbreviations: COMICO, Copenhagen Migraine Cohort; COROCO, Copenhagen Rosacea Cohort; n, number of patients.
