# Supplementary material for: Clinical characteristics of combined rosacea and migraine
Source: Front Med (Lausanne). 2022 Oct 20;9:1026447. doi: 10.3389/fmed.2022.1026447 (PMC9635264; doi:10.3389/fmed.2022.1026447)
Supplement: Supplementary file 5 [file Table_3.pdf]

Supplementary Table 3. Odds ratio in COMICO for patients *with* rosacea compared with patients *without* rosacea.

	Crude OR (95% CI)	Adjusted OR*
**Migraine subtype**		
MO	1	1
MA	1.59 (0.71 – 3.81)	1.84 (0.80 – 4.67)
Both MO & MA	1.02 (0.61 – 1.74)	0.99 (0.58 – 1.70)
**Severity**		
Episodic	1	1
Chronic	1.01 (0.63- 1.63)	1.00 (0.63 – 1.60)
**Migraine attack in the past year**		
1 – 5	1	1
6 – 12	2.50 (0.54 – 17-46)	2.29 (0.33 – 21.34)
13 – 24	1.17 (0.19 – 13.93)	1.68 (0.18 – 13.95)
25 – 36	0.51 (0.06 – 2.88)	0.51 (0.06 – 3.01)
More than 36	0.61 (0.09 – 2.88)	0.65 (0.09 – 3.15)

* OR was adjusted for: age, sex and smoking**.**

Abbreviations: CI, Confidence interval; COMICO, Copenhagen Migraine Cohort; MO, Migraine Without Aura; MA, Migraine With Aura; OR, odds ratio.
