# Supplementary material for: Clinical characteristics of combined rosacea and migraine
Source: Front Med (Lausanne). 2022 Oct 20;9:1026447. doi: 10.3389/fmed.2022.1026447 (PMC9635264; doi:10.3389/fmed.2022.1026447)
Supplement: Supplementary file 6 [file Table_4.pdf]

Supplementary Table 4 Overview of DLQI and rosacea severity in each cohort.

		Rosacea in COROCO			Rosacea in COMICO		
		**All** (n=300)	**Women** (n=203)	**Men** (n=97)	**All** (n=196)	**Women** (n=177)	**Men** (n= 19)
**DLQI**	No impact (0-1), n (%)	128 (42)	74 (37)	55 (56)	115 (58)	100 (56)	15 (79)
	Small impact (2-5), n (%)	102 (34)	71 (35)	31 (32)	54 (28)	51 (29)	3 (16)
	Moderate impact (6-10), n (%)	36 (12)	30 (15)	6 (6)	22 (11)	21 (12)	1 (5)
	Very large impact (11-20), n (%)	33 (11)	27 (13)	5 (5)	5 (3)	5 (3)	0 (0)
	Extremely large impact (21-30), n (%)	1 (1)	1 (1)	0 (0)	0 (0)	0 (0)	0 (0)
	DLQI, mean (SD)	3.6 (4.4)	4.2 (4.7)	2.4 (3.6)	2.3 (3.1)	2.4 (3.1)	1.2 (2.3)
**Severity of rosacea**							
**RASI Severity**	Clear/almost clear, n (%)	53 (18)	44 (22)	9 (9)	44 (22)	40 (23)	4 (21)
	Mild, n (%)	76 (25)	51 (25)	25 (26)	107 (55)	94 (53)	13 (69)
	Moderate, n (%)	140 (47)	95 (47)	45 (46)	38 (19)	37 (21)	1 (5)
	Severe, n (%)	31 (10)	13 (6)	18 (19)	7 (4)	6 (3)	1 (5)
	Severity, mean (SD)	11.9 (5.9)	11.1 (5.6)	13.4 (6.2)	10.3 (4.9)	10.3 (4.7)	9.5 (6.9)

Abbreviations: COMICO, Copenhagen Migraine Cohort; COROCO, Copenhagen Rosacea Cohort; DLQI, Dermatology Life Quality Index; n, number of patients; RASI, Rosacea Area and Severity Index.
