# Supplementary material for: Clinical characteristics of combined rosacea and migraine
Source: Front Med (Lausanne). 2022 Oct 20;9:1026447. doi: 10.3389/fmed.2022.1026447 (PMC9635264; doi:10.3389/fmed.2022.1026447)
Supplement: Supplementary file 7 [file Table_5.pdf]

**Supplementary Table 5.** Odds ratio for COROCO for patients *with* migraine compared with patients *without* migraine in COROCO.

| | Crude OR (95% CI) | Adjusted OR* |
|---|---|---|
| **Rosacea subtype** | | |
| ETR | 1 | 1 |
| PPR | 0.74 (0.44 – 1.23) | 0.63 (0.36 – 1.11) |
| Ocular rosacea | 2.35 (1.46 – 3.82) | 2.61 (1.55 – 4.45) |
| **Flushing attacks in the past year** | | |
| 0 | 1 | 1 |
| 1-12 | 2.65 (1.19 – 6.07) | 1.68 (0.70 – 4.10) |
| Monthly | 2.35 (0.94 – 6.01) | 1.76 (0.65 – 4.82) |
| Weekly | 3.31 (1.54 – 7.35) | 2.00 (0.86 – 4.71) |
| Daily | 4.32 (2.00 – 9.70) | 2.23 (0.96 – 5.31) |

*OR was adjusted for: age, sex, and smoking.

**Abbreviations**: CI, Confidence interval; COROCO, Copenhagen Rosacea Cohort; ETR, Erythematotelangiectatic Rosacea; OR, Odds ratio; PPR, Papulopustular Rosacea.
