# Supplementary material for: Clinical characteristics of combined rosacea and migraine
Source: Front Med (Lausanne). 2022 Oct 20;9:1026447. doi: 10.3389/fmed.2022.1026447 (PMC9635264; doi:10.3389/fmed.2022.1026447)
Supplement: Supplementary file 8 [file Image_1.pdf]

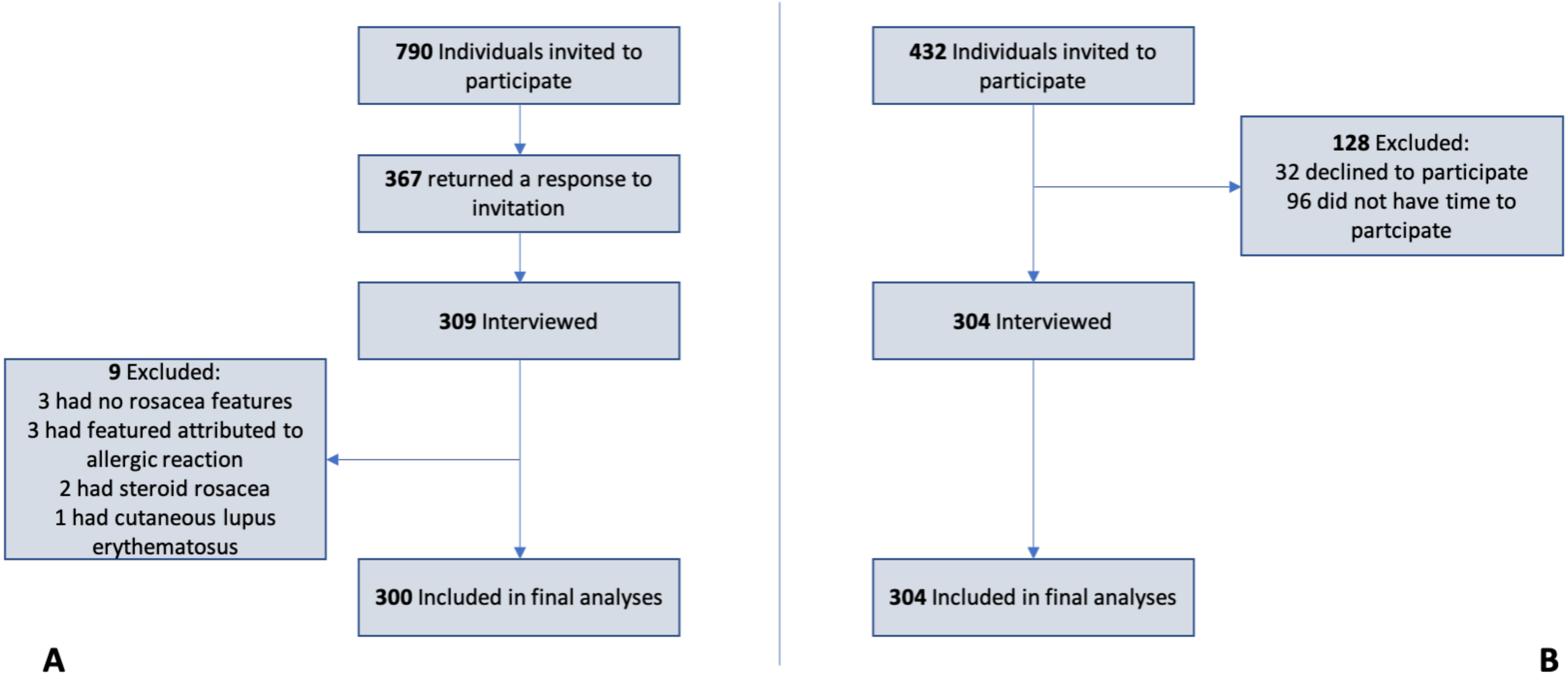

Supplementary Figure 1. Enrollment process for A, Copenhagen Rosacea Cohort (COROCO); B, Copenhagen Migraine Cohort (COMICO).
