# Supplementary material for: Clinical characteristics of combined rosacea and migraine
Source: Front Med (Lausanne). 2022 Oct 20;9:1026447. doi: 10.3389/fmed.2022.1026447 (PMC9635264; doi:10.3389/fmed.2022.1026447)
Supplement: Supplementary file 9 [file Image_2.pdf]

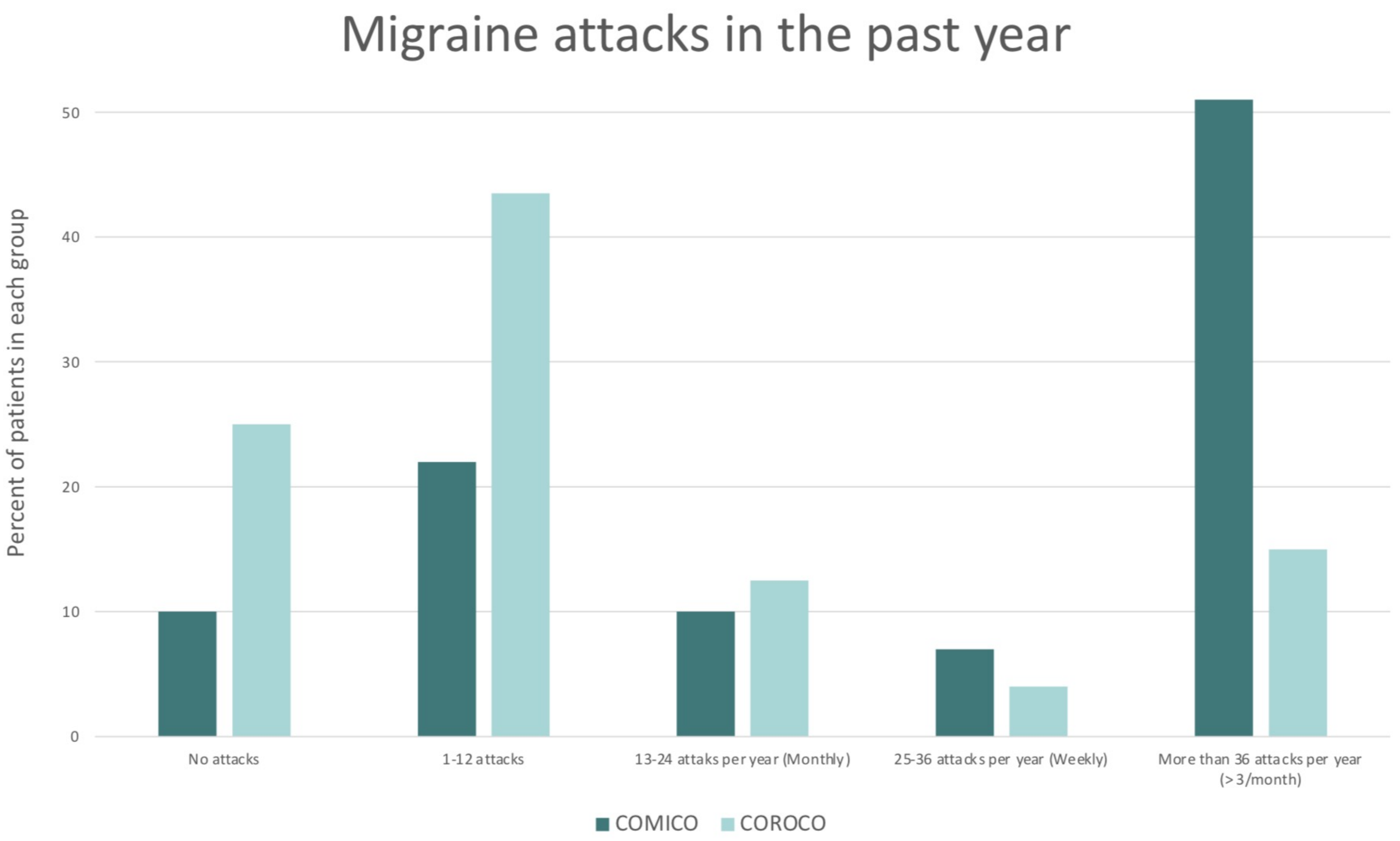

Supplementary Figure 2. Self-reported frequency of migraine attacks per year in those who fulfilled criteria for migraine according to interview for each cohort. COROCO, Copenhagen Rosacea Cohort; COMICO, Copenhagen Migraine
